# Nitrates and Glucosinolates as Strong Determinants of the Nutritional Quality in Rocket Leafy Salads

**DOI:** 10.3390/nu6041519

**Published:** 2014-04-14

**Authors:** Marina Cavaiuolo, Antonio Ferrante

**Affiliations:** Department of Agricultural and Environmental Sciences, Università degli Studi di Milano, via Celoria 2, Milano 20133, Italy; E-Mail: antonio.ferrante@unimi.it

**Keywords:** rocket, glucosinolates, nitrates, antioxidants, cancer

## Abstract

Rocket is an important leafy vegetable crop and a good source of antioxidants and anticancer molecules such as glucosinolates and other sulfur compounds. Rocket is also a hyper-accumulator of nitrates which have been considered for long time the main factors that cause gastro-intestinal cancer. In this review, the content of these compounds in rocket tissues and their levels at harvest and during storage are discussed. Moreover, the effect of these compounds in preventing or inducing human diseases is also highlighted. This review provides an update to all the most recent studies carried out on rocket encouraging the consumption of this leafy vegetable to reduce the risk of contracting cancer and other cardiovascular diseases.

## 1. Introduction

“Rocket” refers to a widely consumed leafy vegetable belonging to the *Diplotaxis* and *Eruca* genera of the *Brassicaceae* family. In the last two decades, rocket has become very popular and widely cultivated, and its production as baby leaf crop steadily increased in the fresh-cut industries. Rocket is a low calories vegetable and its leaves are predominantly eaten raw as a garnish or mixed with other leafy salads. Eating the fresh raw material avoids the use of cooking treatments that would otherwise promote high losses of nutrients and other healthy compounds. Since rocket is a fast and cool-wheatear growing crop, with a sowing time either in spring or autumn, it can be found in the markets throughout the year. Moreover, this crop can be successfully cultivated in hydroponics and greenhouses and can be harvested from regrowth. After the harvest of baby leaves, which occurs after 20 to 30 days, rocket has 14 days in storage and shelf-life, a relatively long time respect to the postharvest life of other leafy vegetables. The postharvest quality is preserved using modified atmosphere controlled packages with low O_2_ and high CO_2_ [1].

Despite the existence of many species that could be potentially used as vegetable crops, *Eruca sativa* L. (salad rocket or annual garden rocket) and *Diplotaxis tenuifolia* L. (wild rocket or perennial wall rocket) are largely used for human consumption. *E. sativa* is widely cultivated in the Middle East and South Asia, while *D. tenufolia* is commonly cultivated and consumed in Europe. *E. sativa* species has white flowers, lobular shaped leaves and is naturally diffused as weed in corn and flax fields, waste places and roadsides [2]. The oil extracted from the seeds is mostly used as lubricant and for soap production [3]. On the contrary, *D. tenuifolia* is naturally present in both uncultivated and cultivated areas on sandy and calcareous soils, roadsides, neglected areas and rock crevices [2]. It has yellow flowers and serrated leaves. Successfully cultivated in uncongenial and hostile environments, wild rocket is a presumed halophyte plant and can potentially be used as vegetable crops for saline agriculture [4].

Commonly characterized by the pungent taste and the strong flavor of their edible leaves, *E. sativa* and *D. tenuifolia* species differ in leaf morphology and seed size, carbon fixation system, growth rate and glucosinolates (GSLs) content [5].

Since ancient times, rocket has being used for many purposes ranging from food to cosmetic and medicinal uses. Diuretic, stimulant, depurative and stomachic activities were recorded and phytochemistry analyses revealed an high content of health-promoting compounds in leaves and seeds, mainly antioxidants and glucosinolates [6,7,8,9] with proven pharmaceutical and anti-cancer properties. 

This review describes the nutrient and anti-nutrient composition of *E. sativa* and *D. tenuifolia* in terms of antioxidants, glucosinolate and nitrate content and how their levels change over environmental and storage conditions. Their beneficial and/or harmful effects on human health are also discussed.

## 2. The Glucosinolates

Glucosinolates are nitrogen (*N*) and sulfur (*S*)-rich anionic secondary metabolites otherwise known as b-thioglucoside-*N*-hydroxysulfates, *cis*-*N*-hydroximinosulphate esters or *S*-glucopyranosyl thiohydroximates [10]. A huge number of genes involved in the biosynthetic pathway of glucosinolates were discovered in *Arabidopsis thaliana* L. [11] and their homologous genes were identified in *Brassica rapa* L. [12].

GSLs share a central core structure holding a sulfonated aldoxime moiety (sulfur side chain) and a variable aglycone moiety (side chain R) that derives from different types of amino acid precursors; according to the origin of the R-group, GSLs are distinguished in aliphatic, aromatic and indolyl [13].

**Figure 1:**
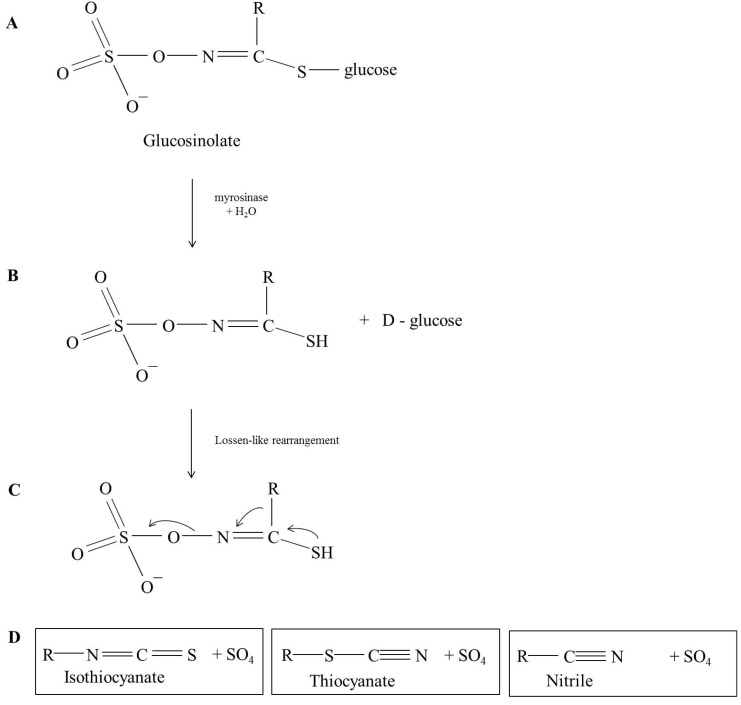
Myrosinases catalize the hydrolysis of glucosinolates (**A**) yielding d-glucose and aglycone (**B**); Depending on the physiological conditions, the aglycone undergoes a Lossen-like rearrangement (**C**) releasing sulfate and isothyocianates, thyocianates or nitriles (**D**).

Glucosinolates are localized in the plant vacuole [14] and are hydrolyzed by myrosinase enzymes, which are β-thioglucoside glucohydrolase (TGG) (EC 3.2.3.1) present in the plant cell and in the human intestinal flora. Upon tissue disruption and cell breakage resulting from plant injury or chewing, TGGs deglucosylate GSLs through the hydrolysis in β-d-glucose and aglycone fragment products [15], whose rearrangement give rise to isothiocyanates (ITCs), thiocyanates, nitriles and sulfates (Figure 1). All these compounds along with GSLs are responsible for the peculiar sharp taste and smell of *Brassicaceae* leafy vegetables [16].

### Glucosinolate Profile and Their Breakdown Products in Rocket

In the last decade, several GSLs were isolated from the seeds, sprouts and fresh-cut leaves of various rocket species [17]. Table 1 summarizes the GLSs found in *E. sativa* and *D. tenuifolia*. The glucosinolate profiles are highly variable but in both species, aliphatic GSLs constitute more than the 80% of total GSLs content. Glucoraphanin, glucoerucin and dimeric 4-mercaptobutyl-GLS are consistently present in high amounts in the leaves [17,18], while seeds and roots contain predominantly glucoerucin, and flowers contain glucosativin [19]. The glucosinalbin is only present in the roots of *D. tenuifolia* but not in *E. sativa* [19]. In *E. sativa* the amount of glucoerucin was registered as 108 ± 5 μmol g^−1^ dry weight DW representing 95% of total GSLs content in the seeds and 79% in the sprouts [9]. In both species, progoitrin/epiprogoitrin and dimeric glucosativin are correlated with bitterness and pungency, while aroma intensity is negatively related to the glucoalyssin content [20].

**Table 1:** List of the glucosinolates found in the two main rocket species.

Chemical name	Common name	Species	Reference
2-Propenyl	Sinigrin	*D. tenuifolia*, *E. sativa*	[21]
3-Butenyl	Gluconapin	*E. sativa*	[22]
Ethyl	Glucolepidin	*D. tenuifolia*, *E. sativa*	[17]
*n*-Butyl	-	*D. tenuifolia*, *E. sativa*	[17]
1-Methylethyl	Glucoputranjivin	*D. tenuifolia*	[17]
4-Hydroxybenzyl	Sinalbin	*D. tenuifolia*, *E. sativa*	[17]
Benzyl	Glucotropaeolin	*D. tenuifolia*	[17]
4-Phenylbutyl	Gluconasturtiin	*D. tenuifolia*, *E. sativa*	[23]
2-Phenylethyl	Gluconasturtiin	*D. tenuifolia*, *E. sativa*	[23]
4-(Methylsulphinyl)butyl	Glucoraphanin	*D. tenuifolia*, *E. sativa*	[17,23]
5-(Methylsulphinyl)-pentyl	Glucoalyssin	*D. tenuifolia*, *E. sativa*	[17,23]
4-(Methylthio)butyl	Glucoerucin	*D. tenuifolia*, *E. sativa*	[17,18,24]
2(*S*)-2-Hydroxy-3-butenyl	Progoitrin/epiprogoitrin	*D. tenuifolia*, *E. sativa*	[25]
2-Hydroxyethyl	-	*D. tenuifolia*, *E. sativa*	[17]
4-Hydroxyindol-3-ylmethyl	4-Hydroxyglucobrassicin	*D. tenuifolia*, *E. sativa*	[18]
Indol-3-ylmethyl	Glucobrassicin	*D. tenuifolia*, *E. sativa*	[18,23,25]
4-Methoxyindol-3-ylmethyl	4-Methoxyglucobrassicin	*D. tenuifolia*, *E. sativa*	[23]
1-Methoxyindol-3-ylmethyl	Neoglucobrassicin	*D. tenuifolia*, *E. sativa*	[20]
4-Mercaptobutyl (dimer)b	DMB-GLS	*D. tenuifolia*, *E. sativa*	[24]
4-(β-d-Glucopyranosyldisulfanyl) butyl)	4-GDB-GLS	*D. tenuifolia*, *E. sativa*	[22]
4-Hydroxybenzyl	Glucosinalbin	*D. tenuifolia*	[19]
4-Mercaptobutyl	Glucosativin	*D. tenuifolia*, *E. sativa*	[24]
4-Pentenil	Glucobrassicanupin	*D. tenuifolia*, *E. sativa*	[23]
3-(Methylsulfanyl)propyl	Glucoiberverin	*D. tenuifolia*, *E. sativa*	[26]

The levels and the chemical forms of GSLs are strongly dependent on the plant species and variety as well as on the environmental, nutritional and growth conditions [27]. Few studies showed that the content of glucosinolates is altered during the storage and under certain pre-harvest and postharvest conditions such as temperature, period of storage, nitrogen sources and light intensity [28,29,30]. In the harvested leaves of *E. sativa* the total GSLs increased up to the third day of storage either at 4 °C or at 15 °C and decreased thereafter [29]. A similar trend was observed in seven-days old seed sprouts in which glucoraphanin and glucoerucin declined after 1 week of storage at 4 °C over a period of 21 days [30]. Among GSLs glucoerucin and glucoiberverin showed a significant reduction during shelf life in both *E. sativa* and *D. tenuifolia*, while only glucoraphanin increased over the storage period. The quantity of 4-(β-d-glucopyranosyldisulfanyl) butyl glucosinolate instead was not affected by variation of light intensity during rocket cultivation [26]. In the work of Selma *et al.* (2010) the use of soil organic amendments such as sewage sludge and urban solid waste in *E. sativa* determined a reduction in the content of total and individual glucosinolates as well as other phenolic compounds, although an increased yield and water content were observed [30].

In *E. sativa* N sources in the form of ammonium (NH_4_^+^) and nitrate (NO_3_^−^) significantly affected the GSLs content: the highest values of GSLs were observed at a NH_4_^+^ NO_3_^−^ ratio of 50/50, while the lowest values were registered in presence of only NH_4_^+^ [31]. In the same species, N fertilizations up to 1.04 g N per plant enhanced growth and biomass during the first month of cultivation with negative effects on the aliphatic GSLs biosynthesis [32]. In contrast, indole GSLs showed a general increase [32]. Additional work is needed to clarify which regulatory mechanisms control the preferentiality towards aliphatic and/or indole GSLs biosynthesis. However, it is clear that N and more in general plant nutrition have an impact on GSLs concentrations by influencing negatively or positively the GSLs biosynthesis and catabolism. A balanced nutrition, therefore, could be useful to optimize the GSLs content.

The isothiocyanates (ITC) are produced at neutral pHs after hydrolysis of glucosinolates. Glucotropaeolin give rise to benzyl isothiocyanate, gluconasturtiin is the precursor of phenethyl isothiocyanate and the hydrolysis of sinigrin releases allyl isothiocyanate. Indole glucosinolates give rise to 3-indolemethanol, 3-indoleacetonitrile, 3,3′-diindolylmethane and indole-3-carbinol [33]. 4-methylthiobutylisothiocyanate (MTBI), also called erucin (ER), derives from the enzymatic hydrolysis of glucoerucin and represents the major ITC in rocket species. Erucin is also generated through the reduction of 4-(methylsulfinyl)butyl isothiocyanate, also known as sulforaphane (SF) analog whose precursor is glucoraphanin. In the work of Jirovetz *et al.* (2002) [34] gas Chromatography-flame Ionization Detector (GC-FID) and gas Chromatography-mass Spectrometry (GC-MS) analyses identified up to 60 components in the solid-phase microextraction headspace from fresh leaves of *E. sativa*. Among them, 4-methylthiobutyl isothiocyanate, *cis*-3-hexen-1-ol, *cis*-3-hexenyl butanoate, 5-methylthiopentyl isothiocyanate, *cis*-3-hexenyl 2-methylbutanoate, and 5-methylthiopentanenitrile were the most abundant ITCs. Finally, iberin is formed from glucoiberin, and was detected in *E. sativa* [35] but not in *D. tenufolia*.

Goitrin is an oxazolidine-2-thione that derives from the spontaneous cyclizing of ITCs containing a hydroxy group in the second position and deriving from 2-hydroxybut-3-enyl glucosinolate [36]. 

At last, only three types of GSLs (allyl-, benzyl- and 4-(methylthio)butylglucosinolates) are known to be degraded in thiocyanates through the *Z*–*E* isomerization of the aglycone [37].

## 3. Nutrients and Antioxidant Compounds

Besides glucosinolates, rocket contains high levels of fiber, nutritionally important minerals and secondary metabolites like ascorbic acid, flavonoids and carotenoids, which are collectively called antioxidants. Plant-derived antioxidant compounds possess free-radical scavenging activities and are highly produced in flowers and leaves in response to oxidative stress and senescence degenerative processes [38]. 

The amounts of proteins and minerals in the leaves and seeds of rocket species were reported in few studies [39,40,41]. Rocket leaves contain higher concentrations of crude fibers, total minerals and carbohydrates while the seeds are more abundant in crude proteins and fats. Among all the elements, Mg, Ca, Fe and K are the most abundant minerals in the leaves, while Ca, Na, P and Cr are prevalent in the seeds [42]. Among carbohydrates in the leaves, glucose was the prevailing sugar representing more than 70% of the total carbohydrates [34]. 

Among the *Brassicaceae* leafy vegetables rocket has the highest content of ascorbic acid (AsA) with amounts of ~110 mg 100 g^−1^ in the leaves [2,43]. However, the concentration of AsA is affected by sowing time and harvesting conditions, particularly by light and temperature. Indeed, several works showed that the content of AsA is greater in fall sowing time rather than spring sowing time with values registered as 58.13 mg 100 g^−1^ DW and 57.41 mg 100 g^−1^ DW [44,45]. This is in agreement with the works of Francke *et al.* (2004) [46] and Fraszczak *et al.* (2006) [47] where shorter days and lower temperature typical of fall season determined a higher content of AsA.

Seeds, roots, leaves and flowers of *E. sativa* and *D. tenuifolia* contain different flavonoids profiles [19]. Polyglycosilated flavonoids were quantified in all rocket tissues except roots and were constituted by high levels of simple and acylated mono-, di-, and triglycosides. Quercetin derivatives were the principal compounds in *D. tenuifolia* leaves and the total flavonoids content ranged from 4.68 to 19.81 g kg^−1^ DW [20]. Quercetin methoxycaffeoyl triglucoside, quercetin caffeoyl triglucoside, dimethoxycaffeic and dicaffeoyl were isolated for the first time in wild rocket and identified as new quercetin triglucoside derivatives [48]. In contrast, kaempferol derivatives were the major group of phenolics present in *E. sativa* leaves ranging from 8.47 to 26.0 g kg^−1^ DW (77%–88% of total phenolics): kaempferol-3,4-diglucoside was the main flavonoid (8.07–23.68 g kg^−1^ DW) followed by isorhamnetin-3,4-diglucoside (1.07–4.75 g kg^−1^ DW) representing the 9% and 16.3% of total phenolics respectively [20]. However, three quercetin were isolated from the leaves of *E. sativa* and identified as quercetin 3,3′,4′-tri-*O*-β-d-glucopyranoside, quercetin 3′-(6-sinapoyl-*O*-β-d-glucopyranosyl)-3,4′-di-*O*-β-d-glucopyranoside, and quercetin 3-(2-sinapoyl-*O*-β-d-glucopyranosyl)-3′-(6-sinapoyl-*O*-β-d-glucopyranosyl)-4′-*O*-β-d-glucopyranoside [49].

Furthermore, quercetin triglucoside and monosynapoyl triglucoside were identified in *E. sativa* seeds with probable roles in germination or defense [50].

Phenolics were susceptible to environmental conditions at both pre and post-harvest stages: quercetin, isorhamnetin, and cyaniding showed a 15-fold increase in their levels under high light conditions and cyanidin showed an increment during shelf life [26]. 

Among carotenoids, lutein and b-carotene were the most predominant. Lutein counted 5.82 ± 0.51 mg 100 g^−1^ fresh weight fresh weight FW in wild rocket and 7.44 ± 0.78 mg 100 g^−1^ FW in salad rocket while b-carotene counted 7.01 ± 1.04 mg 100 g^−1^ FW in wild rocket and 7.96 ± 1.43 mg 100 g^−1^ FW in salad rocket [51]. The levels of xanthophyll pigments varied between 2.46 ± 0.61 mg 100 g^−1^ FW (VAZ) to 0.06 ± 0.01 mg 100 g^−1^ FW (zeaxanthin) in *E. sativa* and between 1.15 ± 0.34 mg 100 g^−1^ FW (VAZ) to 0.05 ± 0.01 mg 100 g^−1^ FW (zeaxanthin) in *D. tenuifolia* [50]. 

Among all pigments, chlorophyll was the most abundant with levels ranging from 359.62 ± 48.16 mg 100 g^−1^ FW in *E. sativa* to 303.23 ± 36.67 mg 100 g^−1^ FW in *D. tenuifolia* [50]. β-Cryptoxanthin, violaxanthin and neoxanthin were also quantitatively identified in *E. sativa* [34]. The concentration of tocopherol, and phytosterol in *Eruca sativa* oils were reported to be 656 mg kg^−1^ and 6.60 mg g^−1^, respectively [51], while no value were reported so far in *D. tenuifolia*. The work of Brock *et al.* (2006) [52] suggested the presence of tropane alkaloids as secondary compounds: calystegines (nortropane alkaloids deriving from pseudotropine) were found in *D. tenuifolia* and calystegine B3 was found in higher concentration.

Besides alkaloids, glycosides, flavonoids, phenolics, Asa, saponins and tannins, phytochemical analysis revealed the presence of the 6.02% ± 0.5% moisture, 27.67% ± 1.8% oil, 29.83% ± 0.8% protein, 2.60% ± 0.5% ash, 3.09% ± 0.4% carbohydrates and 1.60% ± 0.7% fiber in the seeds of *E. sativa* [53]. Gas chromatographic (GC) analyses indicated the composition in essential and non-essential fatty acids in the oil, where erucic acid counted for the 51.2%, oleic acids for the 15%, *cis*-11-eicosenoic acid methyl ester for the 12.5% and linoleic acid methyl ester for the 6.9%) [52]. By means of atomic absorption spectrophotometric analysis Flanders and Abdelkarim (1985) [54] reported high levels of calcium and potassium with value of 1186 mg 100 g^−1^ and 1116 mg 100 g^−1^ of whole seed, respectively. The presence of nutrients and fatty acids suggest that seeds can be used as food or for feed purposes. Finally, the antioxidant properties of *Eruca sativa* were investigated by measuring the 1-diphenyl-2-picrylhydrazyl (DPPH) scavenging activity in callus, *in vitro* shoots, plantlets, and field-derived plantlets indicating the highest levels of radical scavengers in regenerated tissues [55]. 

## 4. Rocket Compounds with Protective Roles on Cancer and Human Diseases

The positive and beneficial effects on human health of the phytochemicals contained in rocket were reported by several clinical research studies. Glucosinolate, isothyocianate and antioxidant compounds are not only able to defend rocket plants from herbivores, insects and microorganisms but also to protect against higher animal degenerative diseases through the regulation of carcinogen-metabolizing enzymes [56,57,58,59,60,61,62,63,64,65,66,67,68,69,70,71,72,73,74,75]. In particular, ITCs induce the activity of phase II drug-metabolizing enzymes [9] and sulforaphane stimulates the detoxification of electrophiles protecting against oxidative stress [57,58,59,60].

Antimicrobial activities of solvent extracts from leaves, roots and seed oil of *E. sativa* were reported on Gram-negative (*Escherichia coli*, *Pseudomoms aeruginosa*, *Shigella flexneri*, *Salmonella typhi*, *Klebsiella pneumonia*) and Gram-positive (*Staphylococcus aureus*, *Staphylococcus epidermidis*, *Bacillus subtilis*) bacteria [53]. The seed oil exhibited the highest inhibition rate for both Gram-positive and Gram-negative, though *K. pneumonia* and *S. epidermidis* was less sensitive. This inhibition was due to erucic and oleic acids as well as to some isothiocyanates like bis-isothiocyanatobutyl-disulphide. Another example of antimicrobial compound comes from *D. tenuifolia* where erucin was found to be effective against pathogenic postharvest fungi [59]. Finally, other studies revealed that the constituents of both *E. sativa* and *D. tenuifolia* seeds act as fumigants against the stored-product insects *O. surinamensis*, *R. dominica* and *S. oryzae* [60].

In general, *E. sativa* showed anti-ulcer, anti-inflammatory, hepatoprotective and anti-cancer activities. For example, leaves extracts reduced the basal gastric acid secretion, the titratable acidity and the gastric ulceration induced by ethanol, indomethacin and hypothermic stresses in pylorus-ligated rats [61]. Whole plant extracts reduced the benzo(a)pyrene-induced genotoxicity in human hepatoma (HepG2) cells through the interaction of erucin, sulforaphane, erysolin and phenylethyl isothiocyanate [62]. *E. sativa* seed (EES) oil was shown to modulate the oxidative stress associated with diabetes mellitus in alloxan-treated rats: 0.06 mL kg^−1^ of oil improved both the hyperglycemia and lipid profile, by regulating the levels of glutathione, malondialdehyde and 4-hydroxynonenal [63]. Seed solvent extracts reduced the growth of melanoma cells and angiogenesis in mice and seed ethanolic extracts displayed a protective roles against mercuric chloride (HgCl2) mediated renal toxicity in rats and against carbon tetrachloride (CCl4) induced hepatic injury in liver rats [64,65]. Again, EES eliminated the negative effects of aflatoxin B1 in the male rat re-establishing the blood, liver and kidney conditions to normal [66]: low doses of EES caused the higher proliferation of haploid cells and an increased mitotic activity that resulted in the stimulation of spermatogenesis in male rat [67].

The work of Melchini *et al.* (2009) demonstrated the anti-proliferative effects of the erucin extracted from *E. sativa* and *D. tenuifolia* on human lung carcinoma A549 cells [68]. By enhancing the expression of PARP-1 cleavage, p53 and p21 that are involved in apoptosis and cell cycle arrest, erucin reduced the proliferation of A549 cells.

Both erucin and sulforaphane were shown to be involved in the prevention and in the therapy of psoriasis and inflammatory skin diseases through the down-regulation of psoriasis-related cytokines, interleukin (IL)-12/23p40, IL-6 and tumor necrosis factor (TNF)-α [69,70].

Despite ITCs exerting the major role as anti-cancer compounds, other rocket derived phytochemicals were reported to have similar functions. For example, the polyphenol extracts from *D. tenuifolia* were shown to exert cytotoxic and anti-proliferative effects on human colon carcinoma (Caco-2) cells [71]. Fifty and 100 mL L^−1^ of polyphenol extracts reduced Caco-2 cell viability up to 71% and 29% respectively. Furthermore, the number of Caco-2 cells treated with 10 mL L^−1^ of these extracts were highly reduced in the S and G2 + M phases. 

## 5. Nitrates in Rocket and the Effects on Human Health

Nitrates are nitrogen compounds largely abundant in nature with high amounts in soil, vegetables and water and as such, they are very important for the food chain of all living organisms. Nitrates are the main source of nitrogen in plants and are indispensable for the biosynthesis of proteins and nucleic acids [72]. Hence, it is important to provide exactly the amount of nitrogen required in order to reach the commercial development stage of leafy vegetables. Nitrates are highly accumulated in the vacuole, where they exert functions in the osmotic regulation especially in absence of other important osmoregulators (e.g., sugars, sodium, chloride). At low light intensity conditions such as cloudy days or winter periods photosynthesis is lowered and nitrate accumulation increases. Among leafy vegetables rocket is considered to be an hyper-accumulator of nitrates as it contains the highest amount of nitrates: rocket can overcome the amount of 9000 mg kg^−1^ FW in different period of the year and in different growing systems [73,74].

Vegetables, water and meat constitute the main sources of nitrate intake in the human diet [75]. However, if present in high amounts, nitrates may have negative effects on human health raising several alarms in the leafy vegetable production and commercialization line. After ingestion, about 5% of dietary nitrate undergoes enterosalivary circulation and is converted in nitrite by salivary enzymes and oral bacteria such as the Gram-positive anaerobes *Staphylococcus sciuri* and *Streptococcus intermedius*; during digestion and in presence of very low pH (hydrochloric acid) nitrates and nitrites undergo further modifications leading to the formation of nitrous acid forms. Nitrous acid can spontaneously turn in many nitrogen oxide compounds such as nitric oxide (NO), nitrogen dioxide and dinitrogen trioxide which can react directly as signals or can associate with other metabolites forming ethyl nitrite, *S*-nitrosothiols, *N*-nitrosamines, nitroalkenes, all with different systemic effects on human body [76]. Nitrite can also react with ammine derived from meat digestion generating nitrosamines, mutagenic compounds that cause neoplasia [77]. More than 300 nitrosamines and *N*-nitrose compounds were shown to have carcinogenic effects on laboratory animals [77] and many experiments demonstrated that about 85% of nitrosamine and 92% of *N*-nitrose compounds were carcinogenic in more than 40 animal species [78]. However, the significance of these effects on human health is still uncertain.

**Figure 2:**
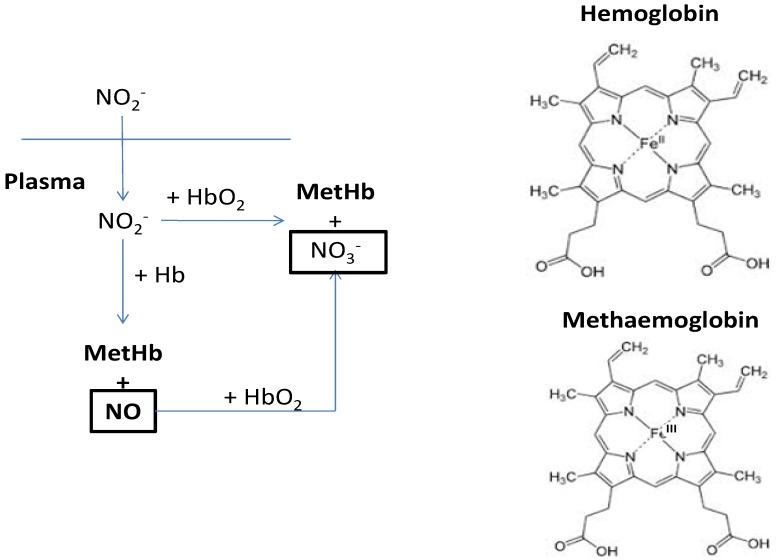
Methaemoglobin (MetHb) formation pathways. The nitrite (NO_2_^−^) oxidizes hemoglobin (Hb), and produces nitrogen oxide (NO) and MetHb. The NO can react with deoxyhemoglobin (HbO_2_) with formation of MetHb and NO_3_^−^. NO_2_^−^ reacting with HbO_2_ can produce MetHb and NO_3_^−^.

Nitrite can also react with hemoglobin to form methaemoglobin and nitrate through the oxidation of iron Fe(II) to Fe(III) (Figure 2). Methaemoglobin is unable to bind and carry oxygen in the blood, a syndrome called methaemoglobinaemia or “blue baby syndrome”. This disease is particularly dangerous for infants up to 3 months of age, but can also affect children and adults, leading to cyanosis and then suffocation [79]. Anoxic tissues with high concentration of methaemoglobin can be visible in the periphery of vascularized organs such as nose and fingers [79]. 

Because of the lack of substantial epidemiological researches [80] many studies reject a direct correlation between nitrate content in food and cancer incidence, highlighting rather the positive aspects of nitrogen compounds on the cardiovascular and immune system [81]. Indeed, NO, *N*-nitrosamines and other secondary reaction compounds produced in the stomach were shown to exert antimicrobial roles against gastrointestinal pathogens such as *Yersinia* and *Salmonella* [82,83]. NO compounds were also shown to have vasodilating and tissue-protective properties as well as modulator activities on platelet, gastrointestinal motility and microcirculation [84]. Finally, nitrate and nitrites as well as NO compounds are important in the regulation of vascular tone and blood pressure in both health and disease states [85].

Despite the diatribe between the positive or negative effects of nitrate compounds on human health, the European Food and Safety Authority (EFSA) [86] stated that the advantages of vegetables in the diet outweigh the disadvantages, therefore the risks for human health associated with the consumption of leafy vegetables are rather low and epidemiological studies do not provide evidence that nitrates are carcinogenic [87]. 

Nevertheless, the free commercialization of leafy vegetables is subjected to strict limitations. This is because certain subgroups of human population could be at higher risk of contracting cancer when subjected to high levels of dietary nitrate intakes. Hence, in view of these negative effects on human health, the World Health Organization (WHO) defined the acceptable daily intake (ADI) of nitrate in 3.7 mg kg^−1^ body weight per day and of nitrite in 0.07 mg kg^−1^ body weight per day [87]. The European Commission Regulation (EC) N° 1881/2006 [88] established the maximum limits of nitrates content in lettuce and spinach, while the EC N° 1258/2011 [89] introduced limitations also for rocket and cereal-based food (Table 2). These thresholds vary from species to species, period of the year and cultivation environment (open field or protected greenhouse). Rocket, for example, has to be commercialized with a nitrate content below 6000 mg kg^−1^ FW in summer-grown rocket or 7000 mg kg^−1^ FW in winter-grown rocket. 

**Table 2:** Thresholds of nitrates for the commercialization of three leafy vegetables based on European regulation 1258/2011 [89].

Leafy vegetable species	Harvesting period	Limits of NO_3_^−^ (mg kg^−1^ FW)
Spinach (*Spinacia oleracea* L.)		3500
		2500
Frozen/canned spinach		2000
Lettuce (*Lactuca sativa* L.) grown in protected environment or open field.	Harvesting from 1st October to 31st March: - Grown in protected environment - Grown in open field	
		5000
		4000
	Harvesting from the 1st April to 30th September: - Grown in protected environment - Grown in open field	
		4000
		3000
Lettuce type “Iceberg”	- Grown in protected environment - Grown in open field	2500
		2000
Salad Rocket, wild Rocket (*Eruca sativa*, *Diplotaxis tenuifolia*)	Harvesting from 1st October to 31st March	7000
Salad Rocket, wild Rocket (*Eruca sativa*, *Diplotaxis tenuifolia*)	Harvesting from the 1st April to 30 September	6000

Strong evidences for either harmful or beneficial effects of dietary nitrates on human health are missing. It appears that nitrates are beneficial at low levels and harmful at intakes above the legal limitations established by EC and WHO. However, many contaminants might be safe at intakes exceeding their limitations. Therefore, extensive researches are still necessary to provide further indications on the dietary nitrates incidences on human health in order to better weigh the potential harmful effects against the benefits arising from the consumption of rocket and other leafy vegetables. This is very important also for farmers, since the application of nitrogen fertilizers on fruit and vegetable crops cause nitrates to remain in the soil and seep out into groundwater making extremely difficult to keep nitrate concentrations below the legal limits.

Different strategies can be used for lowering the nitrate accumulation in rocket leaves during cultivation including the reduction of nitrate fertilization in favor of ammonium form supply, the harvesting time, the use of supplementary light and hydroponic systems with optimized composition of the nutrient solution. Furthermore, monitoring the nitrogen supply in the soil during the growing season of rocket could minimize the risks to exceed the limitation set for NO_3_^−^ content. When the total nitrogen supply (including fertilizer) available in the soil overcomes 200 kg N ha^−1^, the chance to exceed the nitrates limits is very high as well as leaving significant quantities of N residues to leach from soil [90]. 

One of the best strategies to reduce nitrate accumulation was shown to be a rational fertilization consisting in providing rocket with ammonium ions other than NO_3_^−^ [91,92]. This strategy is easily adopted in hydroponics system rather than soil systems where the ammonium is rapidly converted in nitrate. 

Moreover, the nitrate assimilation depends on light and photosynthesis since this provides carbon skeletons (sugars) essential to incorporate the ammonium derived from nitrate reduction and electrons required for the reduction of nitrates in nitrites by nitrate reductase enzyme [93]. Therefore, light intensity and photoperiod increase nitrate assimilation by enhancing the nitrate reductase activity and lowering the accumulation in leaves [93,94]. Furthermore, supplementary lighting was successfully used for lowering the nitrate in spinach and lamb’s lettuce [95,96,97,98,99]. Organic grown rocket usually have higher nitrate content in leaves compared to the conventional ones [99]. In organic cultivation, chemical fertilizers cannot be used and the nutrients supply has to be provided by manure or other organic fertilizers (EEC 2092/91) [100]). However, the mineralization of the organic matter releases the mineral nutrients to satisfy the plant requirements, but during spring and summer, the high temperatures accelerate the mineralization with release of nutrients including high amounts of nitrates. In saline soils, instead, plants have higher osmotic potential by accumulating sodium and chloride ions in the vacuoles [101]. Rocket accumulates less nitrates, since the higher concentration of sodium in the vacuole counteract the nitrate uptake and storage in leaves [102]. The application of environmental stresses during growth of rocket could elicit the accumulation or reduction of certain molecules such as GSLs, antioxidants and nitrates, determining the increment of the overall quality of the final produce during the postharvest storage. High salinity (100 mM NaCl) treatment in hydroponics cultivation of *E. sativa* reduced the nitrate and nitrite contents in the harvested leaves from 2531 mg kg^−1^ DW to 2169 mg kg^−1^ DW and from 2.05 mg kg^−1^ DW to 1.92 mg kg^−1^ DW respectively [103]. Proline applications also reduced the leaf nitrate content from 1527 mg kg^−1^ DW to 1282 mg kg^−1^ DW and nitrite from 2.01 mg kg^−1^ DW to 1.95 mg kg^−1^ DW [104].

All these studies clearly indicated that nitrate content in rocket depends on the adoption of different cultivation systems as well as diverse environmental conditions (Table 3).

**Table 3:** Nitrate content in the leaves of rocket species grown in different environment or cultivation in different agricultural systems.

Species	Nitrate content (mg kg^−1^ FW)	Environment/technique	Reference
*E. sativa*	1575–4139	Organic	[105]
*E. sativa*	2720–6036	Soil	[105]
*E. sativa*	4716–7083	Hydroponics	[105]
*D. tenuifolia*	5300–8100	Peat, vermiculite, and perlite (3:2:1 v/v/v)	[71]
*D. tenuifolia*	2500–3000	Floating system	[73]
*D. tenuifolia*	2524	Soil (grown wild)	[106]
*E. sativa*	1600–3300	Floating system	[107,108]
*E. sativa*	4400	Hydroponically	[28]
*E. sativa*	4000–4500	Floating system	[72]
*E. sativa*	7000	Soil	[72]
*E. sativa*	1282–2531	Floating system	[103]
*E. sativa*	217–341	Soil	[108]
*E. sativa*	432–467	Floating system	[108]
*E. sativa*	500–7200	Floating system	[109]
*E. sativa*	982	Hydroponic Nutrient Film Technique	[110]
*D. tenuifolia*	1859	Hydroponic Nutrient Film Technique	[110]
*E. sativa*	2574	Soil (grown wild)	[106]
*E. sativa*	4700	Peat, vermiculite, and perlite (3:2:1 v/v/v)	[71]

## 6. Conclusions

Consumers choices are very often driven by produce appearance. However, consumers are becoming very much interested in the beneficial effects provided by the produces they eat. In view of these aspects and considering the health benefits of the phytochemicals of rocket, the quick growth cycle and the long postharvest life, we encourage the consumption of this leafy vegetable in combination with other fruits and vegetables. We also believe that these aspects can easily and largely compensate the negative effects of nitrates by respecting the legislation that limit their maximum content in the leaves and daily uptake in the diet in combination with the application of more rationale fertilizations and the use of hydroponic cultivation systems.

Nowadays, rocket breeding programs are aimed at improving the shelf life and the nutritional quality as well as at reducing nitrates accumulation. It would be important to develop new cultivars that in addition to these traits are more tolerant to pre-and postharvest stresses. Still lot of work has to be carried out to understand how the production of phytochemicals and the accumulation of nitrates varies under different pre- and postharvest stresses (e.g., light and temperature) as well as under diverse postharvest technologies in order to obtain cultivars with an higher content of health promoting compounds, reduced accumulation of nitrates and higher resistance to environmental stresses.

The identification and characterization of qualitative and quantitative GSLs and ITCs profiles in other rocket species will help in enriching the range of available rocket species with an higher nutritional value. These species should be also included in breeding programs for their use in the baby leaf salad market along with *E. sativa* and *D. tenuifolia.*

Finally, the pungent taste of rocket leaves is not often appreciated among consumers. Therefore, since flowers are becoming sources of bioactive compounds [34], it would be also worth to investigate better the chemical composition of rocket flowers in order to include them into the diet.
